# The Role of Dietary Nutrients in Male Infertility: A Review

**DOI:** 10.3390/life13020519

**Published:** 2023-02-14

**Authors:** Mona S. Almujaydil

**Affiliations:** Department of Food Science and Human Nutrition, College of Agriculture and Veterinary Medicine, Qassim University, Buraydah 51452, Saudi Arabia; m.almujaydil@qu.edu.sa

**Keywords:** male infertility, semen, sperm mitochondria, dietary nutrients

## Abstract

Male infertility is the main health issue with economic, psychological, and medical attributions. Moreover, it is characterized by an inability to produce a sufficient amount of sperm for the fertilization of an oocyte. Dietary nutrients (DN) have a great effect on male reproductive potential. Observations have indicated that adding DN may protect or treat male infertility. The scope of this criticism is to scrutinize the DN, such as omega-3 fatty acids, vitamins, minerals and other phytochemicals, in enhancing the semen attributes, sperm bioenergetics and sperm functionality in male infertility. It seems that diets rich in omega-3 fatty acids affect sperm quality and maintain the sperm membrane and mitochondria stability. An administration of phytochemicals caused an escalation in sperm mitochondrial function and a decrease in oxidative damage. Furthermore, sundry dietary natural phytochemicals differentially affect (negatively or positively) sperm motility, semen quality, and mitochondrial function, dependent on their levels. Vitamins and trace elements are also nutritional modulators in reducing oxidative stress, thereby enhancing sperm quality, which is accurately connected with sperm mitochondrial function. Also, we described the different types of DN as mitochondrial enhancer for sperm functionality and health. We believe that understanding the DN supports sperm mitochondria and epigenetic modulators that may be responsible for sperm quality and health, and will lead to more embattled and efficient therapeutics for male infertility.

## 1. Introduction

Globally, infertility affects 10–15% of couples, with the male aspect contributing to 20–30% of the cases [1]. Male infertility is a syndrome of the male reproductive function, characterized by the inability to produce a sufficient amount of sperm for fertilization of an oocyte. Male infertility has been disregarded and under-explored as conception was previously observed as a female substance [1]. Moreover, male infertility is a multifaceted phenomenon, and numerous issues include lifestyle, diet, and chronic disease laterally with industrial exposure to certain chemicals, and sperm variables.

In addition, male infertility is frequently instigated by complications in the ejection of semen, sperm production, absence or inferior sperm, sperm kinetics and sperm abnormalities. Commonly, based on the reports of ASRM, 2014, the term “Infertility disorder” is definite as “the incapability to conceive after one year of unprotected intercourse or six months for women aged 35 years or older”. Clinically, infertility is well defined as a couple’s failure to conceive subsequent one year of vulnerable intercourse without pregnancy [2].

**Figure 1:**
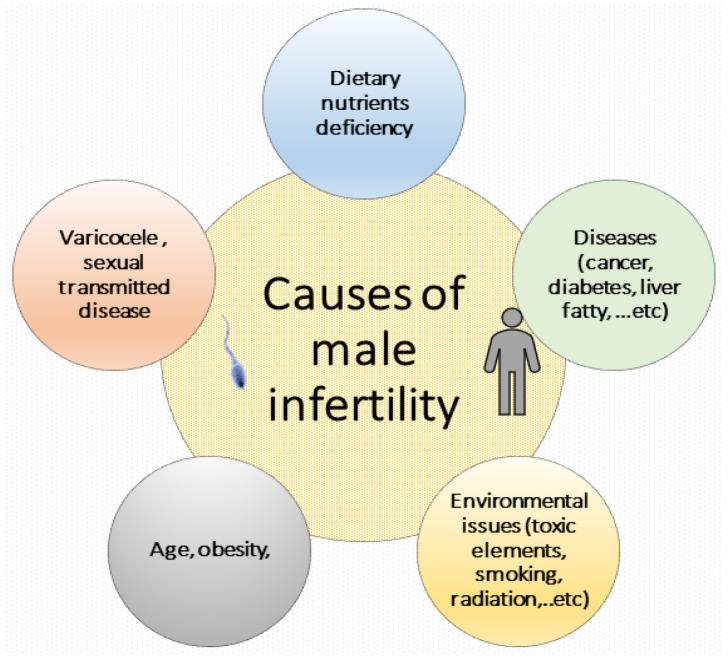
The main causes of human male infertility associated with numerous issues and diseases (cancer, diabetes, liver fatty, varicocele and sexually transmitted diseases), age, obesity, environmental and lifestyle issues (toxic elements, smoking, radiation), and dietary nutrient deficiency.

Every year, scientists observe a huge decline in fertility worldwide [3]. The world Health Organization (WHO) states that there are 45–80 million infertile couples internationally [4]. In the UK, it is assessed that one in seven couples experience complications conceiving, with male-issue infertility accounting for 40–50% of all infertility cases [5]. As referenced by many authors, human male fertility is prejudiced by numerous issues, such as low sperm production, reduced sperm motility, higher sperm abnormalities, medical influences (cancer and pelvic inflammatory illness), non-modifiable influences (e.g., age, gender, and genetics), and utmost significantly adjustable lifestyle issues, e.g., alcohol, nutrition, stress, overweight, physical activity, smoking, and long-term use of preventives. Figure 1 shows the potential effects of previous factors associated with the male infertility.

Additionally, inferior sperm count is the most common cause of infertility, responsible for around 15% of all male infertility cases [6]. Environmental toxins, hormonal disturbances, and genetics could also play a critical role in male infertility [7]. Recently, the association among various biological processes has received more attention in living organisms, especially being influenced by nutrition [8]. Better fertility consequences are noticed in those who are on healthy diets, which comprises supplements with high amounts of phytochemicals, fatty acids, antioxidants and micronutrients [8]. Moreover, it is well accepted that an adequate or balanced diet can be imperative in altering fertility-related consequences in both genders. As it is well known, food is critical for all people to get energy and carry out all indispensable developments, such as reproduction [8]. Providing many active nutrients is essential for all the developmental periods in humans, including growth, puberty, maturity, and reproduction [9]. Following nutritious/well-proportioned food and being physically active are two centers of a healthy lifestyle.; they can sustain a healthy profile and decrease the jeopardy of chronic illnesses [10]. 

In addition, De Rose et al. [11] indicated that misbalanced diets could increase infertility occurrence in humans by 27%. In men viewing low-quality semen, food supplement with micronutrients and antioxidants may enhance sperm superiority, motility, and morphology via mitigating OS-triggered sperm impairment and enhancing hormonal synthesis. Upcoming clinical examinations should be attentive to the conceivable relationship of numerous antioxidants to take benefit of synergistic mechanisms of action. Diet supplements with antioxidant mediators have been reported to reduce male infertility [12]. As previously documented, the high amount of oxidative stress induced by several factors might be responsible for infertility in males. Thus, diet fortifications with antioxidants have exhibited a constructive influence on fertility competence. More randomized controlled studies are desirable to validate the efficiency and safety of micronutrients and antioxidant additions in decreasing male infertility, as well as to discover the optimum dose of each antioxidant and micronutrient to increase fertility competence [9,13]. Results demonstrated a potentially great public interest since fluctuations in diet over the last periods may be part of the clarification for the recently described high incidence of subnormal human sperm amounts. For instance, a decrease in saturated fat uptake may be valuable for well-being and, overall reproductive health. This review aims to epitomize the up-to-the-minute information on dietary nutrient supplements and their relation to reproductive functions and male infertility. In addition, the existing description criticism aims to assess the updates on the functions and mechanisms of action of the dietary nutrients most regularly applied in diet fortifications, as an approach to reduce male infertility disorder [12].

## 2. Research Strategy

A detailed review-literature assessment was achieved to sense all available peer-reviewed documents, which investigated the dietary supplements in improving male infertility and the underlying mechanisms. The exploration was shown via recommended databases, such as Science Direct, Google Scholar, MEDLINE, Scopus and Web of Science. Primary exploration keywords were: male infertility, sperm function, dietary supplements, fat, vitamins, minerals, antioxidants, phytochemicals, and semen quality. The preclinical and clinical investigations and analysis papers from over the last 2–3 decades were involved. Additionally, English language periodicals were included. All applicable investigations were placid and scrutinized precisely to extract relevant facts relating to male infertility and its administration with the dietary supplement. Table 1 summarizes the main effects of some dietary nutrients in treating male infertility.

## 3. Impacts of Lifestyle

As we have depicted in the above data, there are wide-ranging issues that can affect sperm function and health, which results in male infertility. Iatrogenic or hereditary causes are the well-accepted etiologies, and one of the biggest contributors to this failure is modifiable lifestyle influences [44,45,46]. Studies have informed that exposure to numerous lifestyle issues, including smoking, alcohol, obesity, illicit drugs, fast food intake, caffeine and diet, can negatively affects male fertility [47,48]. Uptake of alcohol (>20–25 units per week) had significant adverse effects on spermatogenesis, seminogram and sperm function [48]. Moreover, the drinking of alcohol may be associated with the male infertility by causing the Sertoli-cell syndrome, spermatogenic arrest and antioxidant imbalance due to high levels of seminal leucocytes [48,49]. Additionally, it has been mentioned that chronic alcohol consumption is further associated with epigenetic modulations and the transfer of these modifications to the next generation, which is evolving as a main cause of alcohol-related fetal growth blemishes via changed paternal DNA methylation [46]. In this regard, further clarifications are hunted to explore the transgenerational effects on the offspring health at the omics levels. Modern dietary “Westernized” intake is characterized by processed foods, high-energy sugars and sodium, hydrolyzed fatty acids, and high calories, at the same time reduced vegetable and fruit intake, vitamins and herbs [50]. The extreme intake of this diet may induce several metabolic syndromes and with increase the risk of male infertility shown by poorer seminograms. Sweetened dietary intake escalates the risk of asthenozoospermia, possibly arbitrated via increased oxidative stress and insulin resistance induced through obesity and diabetes [51]. Overall, untoward nutritional consumption relates to augmented testicular dysfunction, sperm DNA fragmentation and diminished chromatin condensation [51]. Epigenetic modulation has been described, with transmission to the offspring. Nevertheless, direct causality has not been demonstrated, although this is associated with an increased risk of metabolic disorders, obesity and cancer in the offspring [52]. Additionally, modern dietary intake may induce obesity, which has significantly expanded in the current era, globally. Moreover, obesity is detected together with a global increase in male infertility [51]. Previous reports have evidenced that obesity can impair male fertility potential, comprising inferior semen attributes, hypogonadism, augmented testicular warmth, augmented pregnancy disorders, and decreased live birth proportions [52]. Davidson et al. [53] have reported that obesity-induced epigenetic changes resulted in DNA methylation, sperm RNA modifications, histone acetylation and protamination alterations [51]. However, additional spotlights are required to recognize the intermediaries of obesity-triggered male infertility.

## 4. Dietary Nutrients

### 4.1. Omega-3 Fatty Acids

Fats are included in 30–40% of daily energy consumption in Western countries, and are substantial ingredients of cell membranes. They change the expression of enzymes that are involved in prostaglandin and steroid hormone metabolism, which are very active in maintaining reproductive capacity [53]. Dietary fats are indispensable for human health and have fundamental functions in cellular structure and hormonal synthesis in the human body.

Recently, a meta-analysis review found that a diet with a low fat content significantly reduces testosterone levels in men, with the greatest impact being on Caucasian men [14]. Types of fats have different effects on male reproduction functions, such as saturated fats (SFs), trans fatty acids (TFA) and polyunsaturated fatty acids (PUFAs). Studies on using saturated fat to treat male infertility are conflicting [9]. Previous investigations exhibited a significant correlation between the intakes of increased SFs to deteriorating sperm accounts by 38%. In another investigation, Jensen et al. [15] clarified an association between reduced total semen volume and SFs, indicating no significant influence has been noticed in any other degree of male fertility.

To clarify the relationship between omega-3 fatty acids and sperm quality, [15] showed that Danish men with inferior sperm counts and motility consumed a high level of saturated fat in their diets. It has been indicated that the high ω3FAs significantly improved the testosterone levels in men. Moreover, sperm kinetics, and semen quality were improved in men as a response to ω3FAs dietary inclusion [54,55]. Up to date, no clear studies have been reported to demonstrate the effects of saturated fat on reproductive health. The association between saturated fat, and sperm quality and further reproductive capacity in humans and animals desires added examination, as high saturated fat consumption is often connected with other unhealthier dietary attitudes universally, that may harmfully affect sperm quality and strength. PUFAs are another component of fats. Their intake correlates with an assortment of constructive health consequences, from enhanced blood lipid outlines to mitigation of depression and joint pain [54].

For a long time, it has been supposed that the increasing PUFA consumption is a mutual mediation for enhancing indicators of male fertility. Inappropriately, a robust explanation is required, due to the landscape of dietary scrutiny using FFQ (food frequency questionnaires), indicating that any association delineated cannot evidence interconnection [55]. However, fortification with ω3FAs, such as DHA (docosahexaenoic acid) and EPA (eicosapentaenoic acid), has been considered in organized paths. Results from different studies have evidenced that ω3FAs at levels of 0.5–2 g/day exhibited beneficial influences on sperm kinetics and improved semen quality, thus enhancing sperm health and function, based on meta-analysis reports [17].

Moreover, a double-blind placebo conducted by [55] elucidated that supplementation of 0.5–2 g/day DHA presented a considerable enhancement in sperm motility and minor improvements in oxidative stress indices in infertile men (asthenozoospermia men). Almost 20% of TFAs (trans fatty acids) can be found naturally in products derived from animals, such as dairy and meat products. Industrially combined hydrogenated fats applied during nutriment manufacturing now explains the 80% TFA consumption [16]. Few reports intended a negative association between male fertility and TFA intake via disturbances in testicular function, changes in testosterone levels, and sperm function and quality. In other words, a clear relationship was demonstrated, whereby a positive link between sperm health and TFA uptake exists. It was found that a linear amount connected an inferior sperm function and quality with every augmented quartile of TFA consumed [18]. It was found that the significant negative connection between TFA consumption, sperm quality and fertilization outcomes. However, this work had some different subjects [18].

Moreover, a review by [15] was applied using eight studies, totaling 1355 men. The previous research performed showed that TFA consumption is related to numerous negative health significances, and reduced sperm superiority and health are involved in that decline, but further clarifications and well-designed research may be required to evaluate the men accurately. The limitation of this work is that it utilizes a cohort of 701 young Danish men. Still, it did not discriminate between the potential effects of TFA and saturated fat on sperm health. Furthermore, the increase in the ω-3 in the sperm plasma membrane phospholipids encourages satisfactory antioxidant defense and sustains the plasma structure, decreasing the threat of impairment to sperm cells [55]. An earlier study has found that low levels of ω3FAs in dietary intake may be associated with male infertility by reducing sperm quality and health [19]. PUFAs are precursors to locally synthesized hormones, such as eicosanoids. These signaling molecules show multilayered and serious functions in human health and reproduction [16].

**Figure 2:**
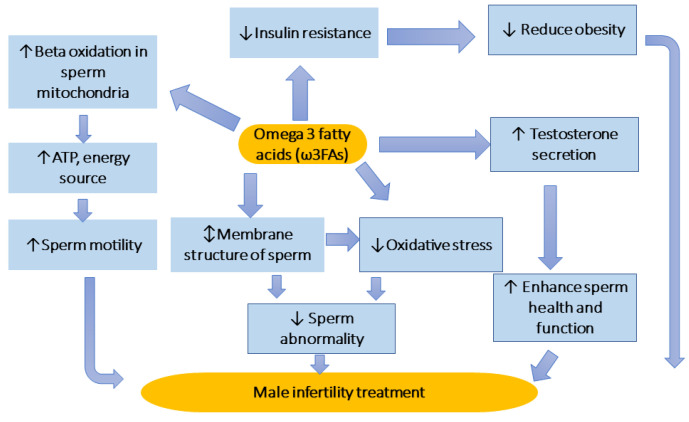
The role of ω3FAs in treating male infertility. ω3FAs dietary intake can enhance antioxidative defense, reduce oxidative stress and βeta-oxidation in sperm mitochondria, sustain sperm membrane, reduce obesity and enhance sperm health and function, thus alleviating male infertility.

DHA and EPA are long-chain PUFAs of ω3FAs, which can be presented in higher levels of marine fish and some green plants, seeds, herbs, nuts, and oils [16,56]. Figure 2 illustrates the role of ω3FAs in treating male infertility. ω3FAs possibly have a strong antioxidant counteraction to the oxidative stress generation, and augment the antioxidative defenses opposing to the oxidative stress [57].

Multi studies have reported a significant association between ω6/ω3FAs and DHA and PUFA amounts in spermatozoa [20,57].

With this sense, [19] evidenced that the addition of idiopathic infertile patients with EPA and DHA (1.84 g/day) for 4 months boosted the defensive system in seminal fluid in a placebo, randomized study. The same authors suggested that the link between diminished ω3FA doses and the augmented oxidative DNA destruction of spermatozoa was described in infertile men, endorsing that insufficient intake of ω3FAs could be involved in infertility (Figure 2). Recently, [58] investigated the efficacy of vitamin E (100 mg) and ω3FA (300 mg/day) addition on reproductive catalogs among employees in an automobile portions industrial vegetable. As several authors indicated, ω3FA dietary intake can enhance the antioxidative defense, βeta-oxidation in sperm mitochondria, reduce oxidative stress, sustain sperm membrane, and enhance sperm health and function (Figure 2), thus alleviating male infertility [20,56,57,58].

They reported that the simultaneous intake of ω3Fas and Vit E had a statistically significant result on sperm motility and morphology. Another mediator discovered to have a potential role in fertility is resolving D1 (RvD1), formed by DHA, which applies pro-resolving and anti-inflammatory actions. [57] showed that the RvD1 levels were connected with decreased sperm quality and higher levels were detected in infertile patients. RvD1 is a novel biomarker for infertile status, indicating lipid peroxidation in seminal fluid. Previous research proposes a panel of inflammatory pointers and lipid intermediaries for a diagnosis of inflammatory-grade and a suitable therapeutic method. Owing to its significant functional and protective role, the preclinical and clinical trials are indispensable to elucidate the advantageous impacts of ω3FAs on infertile cases [9].

Likewise, combined therapy with ω3FAs behind chemical agents is recommended. According to the abundant published papers, ω3FA dietary supplementation enhanced male reproductive functions via several pathways, including reduced DNA damages in sperm, better sperm motility, concentration, improvement in testosterone secretion, and regular morphology, and increased seminal antioxidant grade and reduced the proportion of apoptotic damages to sperm cells [57]. These are possible modes of action of the role ω3FAs in treating male infertility, as offered in Figure 2.

### 4.2. Vitamins

**Figure 3:**
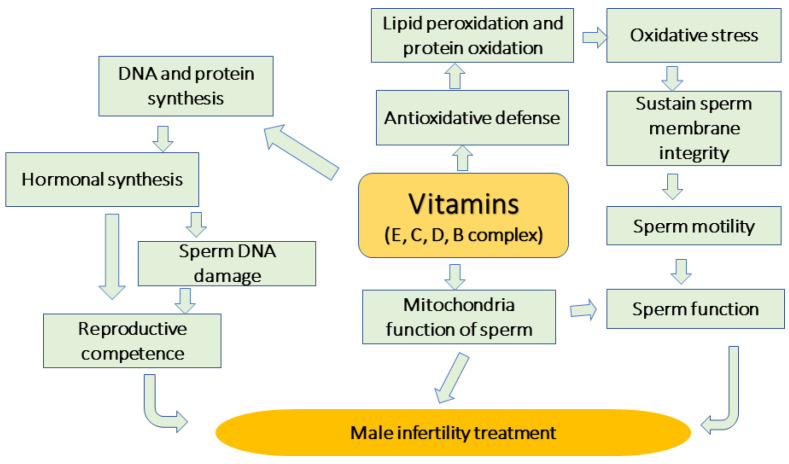
The protective function of some vitamins on sperm function and quality in male infertility. This machinery is caused by reducing lipid peroxidation, sustaining sperm membrane integrity, supporting ATP production in mitochondria, therefore enhancing sperm motility.

Vitamins are organic molecules that are indispensable micronutrients in small quantities for the appropriate functioning of many biological functions in the body. Figure 3 illustrates the role of vitamins in treating male infertility. In this sense, vitamins can support antioxidant ability, sustain sperm DNA, lipid and proteins from damages, and thus reduce the accumulation of oxidative stress, as well as sustain the sperm membrane. Moreover, vitamins could stimulate the b-oxidation in the mitochondria, such as vit. E, which resulted in ATP synthesis, therefore boosting sperm motility, sperm function and improving reproductive competence (Figure 3).

Based on its solubility in water, there are two groups of vitamins: one is soluble in water and the other is soluble in fats. For instance, vitamin E (alpha-tocopherol) is a fat-soluble molecule found in spinach, avocados, sweet potatoes and almonds. Many previous reports clarified its powerful antioxidant activities by neutralizing free radicals and constraining ROS destruction to cell membranes, resulting in the avoidance of lipid peroxidation and augmentation of another antioxidative system [21,22].

Reports from the European Commission Directive 2008/100/EC have endorsed the daily consumption of 12 mg vitamin E [22]. The association between the decline of vitamin E consumption per day and fertility outcomes has been drawn in animals [32]. However, there are few studies regarding this topic in humans. Moreover, it is interesting to clarify the potential roles of vitamins in treating infertility in humans. As reported in an interventional placebo-controlled trial of infertile men, it was observed that a negligible number of sperm DNA impairments decreased after eight weeks of vitamins E and C supplementation (1 g/day for each one) [59]. Another important vitamin, vitamin C, is a water-soluble vitamin with antioxidant assets, found in fresh berries and citric fruits. An abundance of previous reports have assessed the impacts of vitamin C (vit. C) supplementation on sperm function [22]. An earlier study by Lewis and Simon [60] demonstrated that male rats given vit. C could restore testicular damages triggered by cyclophosphamide. The reduction in vit. C and higher levels of ROS in the seminal fluid of men was associated with asthenozoospermia occurrence, as mentioned before [23]. This could indicate the importance of vit. C in male fertility and reproductive outcomes. In a meta-analysis, [22] reported that vitamins E and C can expand the spousal reproductive competence and semen variables in infertile men without adverse effects. Cobalamin, or vitamin B12, is a water-soluble vitamin that is a co-factor in DNA synthesis, and amino and fatty acid metabolism. After extensive research on the effects of vit. B12 on infertility [33], it has been revealed that vit. B12 has significant effects on sperm function and semen quality, certainly via augmenting sperm motility and sperm account, and diminishing sperm DNA injury [24] (Figure 3). Vitamin D (vit. D) is a fat-soluble vitamin that naturally exists in a few variabilities of foods, so it is essential to add it to foods as a dietary supplement. Chemically, it is also shaped endogenously when ultraviolet rays from daylight trigger vit. D synthesis in the skin.

Many functional properties of vit. D were found, including regulating the Ca and P homeostasis in the body and sustaining bone, muscle and tooth health [61]. In the research of [25], men with infertility (330 individuals) that received vit. D (300,000 IU) and Ca (0.5 g/day) for 5 months had a higher number of spontaneous pregnancies compared to the untreated group, with a difference in sperm amounts. Furthermore, vit. D administration in sub fertile men positively affects semen function and quality via enhancing sperm motility, sperm function, as well as improving the in vitro fertility competence [24,26]. According to scientific reports, most human trials propose that the deficiency of vit. D shows determinable effects on both male and female fertility [27]. Nevertheless, there is still no robust and convincing confirmation from interventional research; utmost trials were of lesser model bulks with a main regard to vit. D intake level and extent. In their study, [34] recommended that vit. D plays a significant function in continuing normal reproductive functions through an impact on sperm count and regular morphology; hitherto its function is not completely based on its antioxidant capability.

A deficiency of vit. D caused an increase in OS markers, which is accountable for changed sperm variables, a significant risk for weakened fertility. Hussein et al. [28] clarified deficiency of vit. D and vitamin D receptor (VitDR) gene methylation might be complicated, by the indicating of male infertility disorder. The same authors suggested that methylation of the VitDR gene was significantly superior in infertility patients compared to normal cases. According to those results, male infertility could be associated with a deficiency of vit. D via epigenetic modification of the VitDR gene and result in gene silencing [28]. Figure 3 exemplifies the protective function of some vitamins on sperm function and quality of male infertility. Within this scope, we need further explorations to clarify the epigenetic modification mediated by male infertility.

Additionally, carotenoids are a group of organic constituents in yellow, red, orange, and pink vegetable dyes, which represent precursors for vit. A, of which the integral constituent is retinol. Carotenoids are robust antioxidants for sperm function, by maintaining cell membrane integrity. As indicated, they are involved in the development and differentiation of sperm during the spermatogenesis process. Carotenoid insufficiency can lead to diminished sperm motility and functionality, resulting in male infertility [21].

Pretreatment of astaxanthin might decrease male infertility by avoiding OS-triggered fertility complaints. However, astaxanthin pretreatment might also defend germ cells alongside MTX-triggered OS [29]. Moreover, astaxanthin is a natural xanthophyll pigment extant in many plants, microbes and other marine organisms, with robust antioxidant action; meanwhile, it is 100–500-fold more active than vitamin E in docking OS and reducing lipid peroxidation [62]. A confident result of astaxanthin on sperm features and fertility competence has been anticipated [63], whereby a molecular source can be elucidated by enhancing mitochondrial function. In detail, astaxanthin seems capable of raising the potential of mitochondrial membrane, functionality and ATP synthesis [8], which are significant events of mitochondrial functionality. The exact mode of action of carotenoids needs further clarification in treating male infertility.

### 4.3. Trace Elements

Trace elements are essential minerals, but are needed in small amounts for the body’s cellular function. One of the most common trace elements is selenium, then Zn, a vital element that naturally exists in foods and should be presented as a dietary complement in global trade. Zn plays significant roles in DNA and protein synthesis, immunomodulatory effect, and enhances cell development and division. In addition, Zn represents robust antioxidant ability via scavenging OS, and can protect the testicular system from damage caused by several environmental issues [64]. However, high amounts of supplemented Zn may increase oxidative stress, inducing mitochondria dysfunction. Many previous reports [22] have indicated a positive association between lower Zn amounts in the seminal plasma of infertile males and the fertility outcomes related to normal males [30]. This pattern could represent treating Zn with importance in fertility, and treating subfertility and infertility.

Furthermore, Zn addition was displayed to promote sperm health in infertile males considerably. Accordingly, this may clarify the significance of this trace element in forming sperm DNA. Nevertheless, these trials had small model sizes and it was not fully understood if the variation in seminal plasma Zn levels was instigated by male infertility syndrome or the same inversely. Iron (Fe) is an important microelement and has a significant function with the circulatory system erythrocytes. A deficiency in Fe in the blood might be associated with several diseases, such as anemia. In a cross-sectional study, [31] investigated the correlation between Fe and semen quality in males (209 healthy Spanish students). They found that a statistically substantial converse association between Fe consumption from the diet and sperm quality health was distinguished. Nevertheless, additional clarifications in male populations referring to infertility issues are desirable. Moreover, Fe consumption was related to fecund ability, where non-heme Fe consumption and complement usage were unreliable, nonetheless roughly suggested valuable impacts on fertility among women suffering from Fe insufficiency [65].

### 4.4. Mitochondria Enhancers

#### 4.4.1. CoQ10

CoQ10 is a constituent of the electron-transport chain accountable for producing ATP particles from aerobic cellular respiration. Moreover, the CoQ10 participates as a robust hunter of free radicals, an energy-stimulating mediator and a membrane stabilizer, which avoids lipid peroxidation [35], suggesting its robust anti-oxidative activity. Previous work in humans found a remarkable relationship between the higher levels of CoQ10 in the seminal plasma and semen health and quality variables (primarily sperm motility and amount). An earlier study indicated a strong negative association between levels of CoQ10 and sperm motility in infertile men [35]. Consequently, this suggests the potential relationship between CoQ10 change and infertility. Besides, Balercia et al. [66] clarified that the lower CoQ10 level was noticed in the sperm with inferior motility and abnormal morphology in men with asthenozoospermia. Kobori et al. [36] found that CoQ10 (120 mg/day) for 3–6 months in infertile patients produced a substantial enhancement in sperm attributes.

**Figure 4:**
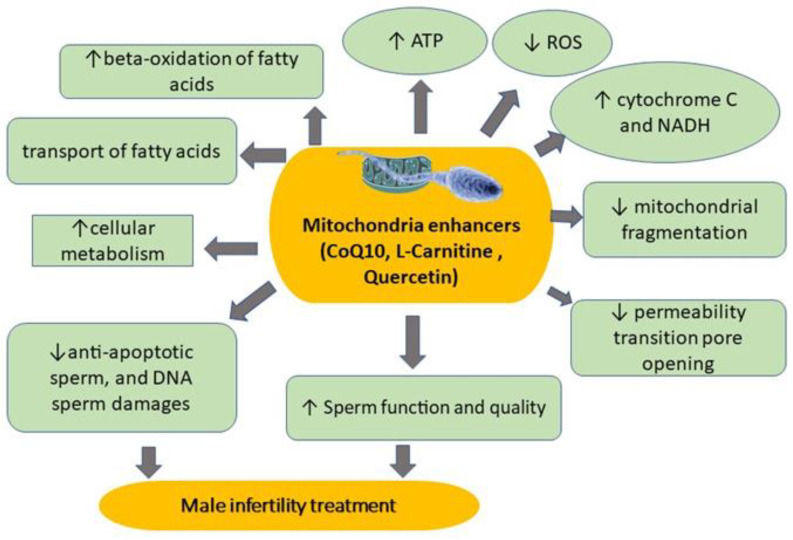
Effects of some natural molecules (CoQ10, L-Carnitine and quercetin) as mitochondrial enhancers in sperm. Those molecules increase fatty acid oxidation in mitochondria, thus increasing cellular metabolisms, such as the production of cytochrome C, NADH, and ATP levels in sperm.

Additionally, it has been found by [37] that the addition of CoQ10 (100 mg/day) in infertile patients for 3 months enhanced the antioxidant status and sperm attributes. Correspondingly, CoQ10 is an applicable, safe antioxidant opposing the numerous pathologic situations, and may enhance sperm function in infertile men. In several animal studies, CoQ10 effectively enhanced the mitochondrial function via boosting the ATP producing and lowering the production of OS in sperm (Figure 4). CoQ10 is able to inhibit cell death, encourage the inhibition of cell death and encourage cell death by hindering mitochondrial fragmentation and the permeability transition pore opening [8]. Contrariwise, further human and animal trials are necessary to authorize its competence against infertility disorder.

#### 4.4.2. L-Carnitine

L-Carnitine (LCN) is a non-protein amino acid (lysine and methionine) with a nutraceutical mediator. This element is an indispensable co-factor for the beta-oxidation of fatty acids in mitochondria and participates in the cellular energy source through the transport of fatty acids from cytosol to mitochondria [67].

High levels of LCN were found in human epididymis, which may affect the luminal situation of the testes to improve sperm function and quality [38]. As previously indicated by [38], the accepted LCN levels of sperm can extend the sufficient ATP for sperm function by enhancing cellular metabolism (Figure 4). Conversely, certain publications have confirmed that inferior levels of seminal fluid LCN might be associated with male infertility [39].

Likewise, LCN has protective effects during cryopreservation, as its prevailing antioxidant ability has been established [39], by reducing the germ cell’s apoptosis and increasing the occurrence of sperm motility in rats exposed to testicular radioactivity [68]. This feature could be associated with the potentiality of LCN in enhancing the mitochondrial function. In other research [40,41], LCN oral administration reduced the number of anti-apoptotic sperm and DNA sperm damage, as well as enhanced sperm function and quality. In a study on infertile rats [5], management with LCN (169 mg/day) and meloxicam (12 doses of 0.6 mg/kg) for 3 months considerably reinstated the quantity of testicular Leydig cells. As well, [23] revealed that adding LCN in semen samples, planned for incubation, was shown to restore sperm vitality and quality. Therefore, the efficiency of LCN, a safe and effective mediator, in treating infertility in male humans is established, while more examinations are required to understand the valuable impacts of LCN in treating human infertility.

#### 4.4.3. Quercetin

New antioxidant remedies are continuously developing, and their uses are significant. However, the popularization of natural molecules has prompted the pharmacological trade investigation to be superior in discovering natural molecules. On an international measure, around 50% of pharmaceutic outputs comprise natural molecules, and around 25% of prescription drugs are derived from natural bioactive molecules [69]. Quercetin (QUR) is the most popular flavonoid as a natural bioactive molecule. Through glycosylation, it creates the backbone of numerous other flavonoid compounds. According to each person’s dietary habits, it is existent in a wide range of foods, and daily intake of quercetin by a humans can differ from 0.01 to 0.1 g. Some authors [70] suggested that quercetin uptake can extend up to 0.5–1 g/day due to its superior purified and extracted availability.

Moreover, QUR applies its antioxidant function by promoting the body’s defense system. QUR generally exists in dietary supplements. QUR seems to have a favorable impact on hypertensive individuals by encouraging a blood pressure decrease [71]. In the study of [42], it was clarified that QUR produced a significant protective role opposing OS in rat sperm via enhancing sperm quality and health, and reducing sperm abnormalities. QUR (100 μM, 2 h of incubation) was likewise demonstrated to significantly enhance sperm function of infertile men, where sperm are naturally more disposed to agonize from high levels of OS [43]. The previous trial (Figure 4) also indicated the reduction of sperm mitochondrial DNA impairment, along with an escalation in cytochrome C and NADH amounts in the semen samples of infertile men [43].

Furthermore, the study of [42] displayed that adding quercetin (50 μM) to a freezing extender significantly increases post-thaw human sperm attributes, precisely sperm viability, DNA integrity, motility, and mitochondria function in relation to normal cases. However, the cytoprotective duty of QUR during sperm freezing needs to be discovered. Few authors have discovered the in vivo possibility of quercetin regarding treating male infertility. In addition, it is of interest to discover more potential effects of quercetin in sperm mitochondria as a new mitochondria modulator, to enhance semen quality and improve reproductive outcomes. Animals with diabetes administrated with QUR offered decreased levels of sperm DNA destruction and enhanced sperm antioxidant markers (GPx, CAT and SOD). To confirm the genetic modulating role of quercetin in sperm functionality, augmented levels of *GPx1*, *CAT*, and *SOD1* mRNA expression were observed [72]. More explicitly, QUR also seems to have valuable impacts in the defense of testicular tissues against some toxic elements, such as cadmium lead nitrate [73,74], as well as diabetes in the animal testes [75].

Nevertheless, to our information, the possibility of quercetin as a remedy for other syndromes has not been discovered in other male infertility. Recently, QUR (0.1–1000 nM, Figure 4) prompted the energetic state of mitochondrial respiration, producing the uncoupling between electron transport and ATP formation [8]. This might be ascribed to the ability of quercetin to interact directly with mitochondrial membranes, such as the coenzyme Q-binding spot, destroying OS and driving the synthesis of ATP [76]. Recently, Mousavi et al. [77] clarified that QUR coated with whey protein is a novel biocompatible antioxidant medication in male infertility problems, influenced by the western-style diet. Figure 4 shows some natural molecules, such as CoQ10, LCN and QUR, that are used as mitochondrial enhancers in sperm.

## 5. Conclusions

The frame of this narrative critique was to explore the up-to-date evidence on the impacts of a dietary mediation that comprises ω3FAs, vitamins, minerals, and some phytochemicals, focusing on their intake treating male infertility and thus improving the reproductive potential in males. The machinery of these compounds could be related to their ability to improve the antioxidative status and reducing oxidative stress. Moreover, these molecules may enhance the available energy for sperm motion, enhance mitochondrial function and sustain DNA integrity of sperm, thus enhancing sperm health and function. Whereas only scarce investigations were established to be authorized, the positive impact of those dietary nutrients on main parameters for male fertility is promising and maintained by biological acceptability. This screening emphasizes the importance of interventions for managing and preventing male infertility. More investigations are still required to clarify more precise mechanisms of mitochondrial enhancers to treat male infertility.

## Figures and Tables

**Table 1:** The main effects of some dietary nutrients in treating male infertility.

Dietary Nutrients	Doses	Main Effects	References
Omega-3 fatty acids (ω3FAs)	ω3FAs (0.5–2 g/day)	High ω3FAs significantly improved the testosterone levels in men. Sperm kinetics and semen quality were improved in men as response to ω3FAs dietary inclusion.	[14,15,16]
	DHA (0.5–2 g/day) for 4–12 weeks.	DHA addition presented a considerable enhancement in sperm motility and minor perfections in oxidative stress indices in infertile men (asthenozoospermia men). High level of TFA intake signficantly improved sperm quality and fertilization outcomes.	[11,17,18]
	EPA and DHA (1.84 g/day)	EPA and DHA (1.84 g/day) administered for 4 months boosted the defensive system of seminal fluid in a placebo study. Low levels of ω3FAs in the dietary intake were associated with the occurrence of male infertility through a reduction of sperm quality and health.	[19,20]
	ω3FAs (300 mg/day) and vitamin E (100 mg)	Intake of ω3FAs and vit. E showed a statistically significant improvement in sperm motility and morphology.	
Vitamins	Vit. C	Low levels of vit. C is related to higher ROS in the seminal fluid of men with asthenozoospermia. Deiatry vit. B12 (1.5–6 mg/day) had significant effects on sperm function and health via augmenting sperm motility and sperm account, and diminishing sperm DNA injury. Infertile men (330 individuals) received Vit D (300,000 IU), and Ca (0.5 g/day) for 5 months and had higher sperm fucntion and the number of spontaneous pregnancies in relation to another untreated group. Vit. D administration in sub-fertile men positively affects semen function and quality by enhancing sperm motility, sperm function, as well as improving the in vitro fertility competence. The deficiency of vit. D and vitamin D receptor (VitDR) gene methylation may be complicated, with indicated male infertility disorder.	[21,22,23,24,25,26,27,28]
	Vit. B12		
	Vit. D		
Astaxanthin	Astaxanthin (720 mg/kg body weight)	Pretreatment of astaxanthin (720 mg/kg body weight) might decrease male infertility by avoiding OS-triggered fertility complaints.	[29]
Trace elements		A positive association between the lower Zn amounts in the seminal plasma of infertile males and fertility outcomes in relation to normal males was detected. A statistically substantial converse association between Fe consumption from diet and sperm quality health was distinguished.	[30,31]
Mitochondria enhancers	CoQ10 (100–120 mg/day)	A remarkable relationship between higher levels of CoQ10 detected in seminal plasma and sperm health and quality variables was observed. Supplementation of CoQ10 (120 mg/day) for 3–6 months in infertile patients produced a substantial enhancement in sperm attributes. Dietary therapy of CoQ10 (100 mg/day) in infertile patients for 3 months enhanced the antioxidant status and sperm attributes. CoQ10 can effectively enhance mitochondria function via boosting the ATP production and decreasing the production of OS in sperm.	[8,32,33,34,35,36,37]
	L-Carnitine (LCN)And Quercetin (QUR)	Oral LCN administration reduced the number of anti-apoptotic sperm, and sperm DNA damages, as well as enhanced the sperm function and quality. Management with LCN (169 mg/day) and meloxicam (12 doses of 0.6 mg/kg) for 3 months considerably reinstated the quantity of testicular leydig cells in elder men. QUR (100 μM, 2 h incubation) was likewise shown to significantly enhance the sperm function of infertile men, where sperm are naturally more disposed to agonize from high levels of oxidative stress. QUR addition produced significant reduction in sperm mitochondrial DNA impairment, along with an escalation in the cytochrome C and NADH amounts in the semen samples of infertile men. Adding of QUR (50 μM) to the freezing extender significantly increases post-thaw human sperm attributes, precisely sperm viability, DNA integrity, motility, and mitochondria function in relation to normal cases. Upregulation of *GPx1*, *CAT*, and *SOD1* mRNA expression in sperm as response to QUR administration was observed. QUR (0.1–1000 nM) prompted the energetic state of mitochondrial respiration, resulting in the uncoupling between electron transport and ATP formation.	[5,38,39,40,41,42,43]

## Data Availability

Not applicable.
